# Hair dye poisoning and the developing world

**DOI:** 10.4103/0974-2700.50749

**Published:** 2009

**Authors:** Krishnaswamy Sampathkumar, Sooraj Yesudas

**Affiliations:** Department of Nephrology, Meenakshi Mission Hospital and Research Centre, Lake Area, Melur Road, Madurai, Tamil Nadu - 625 107, India

**Keywords:** Hair dye poisoning, paraphenylene diamine, suicide

## Abstract

Hair dye poisoning has been emerging as one of the important causes of intentional self harm in the developing world. Hair dyes contain paraphenylene-diamine and a host of other chemicals that can cause rhabdomyolysis, laryngeal edema, severe metabolic acidosis and acute renal failure. Intervention at the right time has been shown to improve the outcome. In this article, we review the various manifestations, clinical features and treatment modalities for hair dye poisoning.

## INTRODUCTION

Globally suicide rates have increased by 60% in the 50 years. Suicide is now ranked among the three leading causes of death in the age group between 15 and 44 years. In developing countries pesticide poisoning is one of the leading contributors to this preventable tragedy. Recently, hair dye poisoning is emerging as an important etiological factor. This review examines the pathophysiology and clinical features of hair dye poisoning.

## HAIR DYES-BASIC FACTS

Among the Egyptians, there were hairdressers as early as 5000 years BC and the art of dyeing hair with vegetable dyes was known already at that time. The first artificial dye was synthesized in the laboratory in 1856, and permanent hair colorants have been in commercial use for over 100 years.[1] Hair dyes can be divided into five categories, each with a specific composition and action mechanism: gradual hair coloring (using metallic dyes such as salts of lead, bismuth or silver), vegetable hair dyes (such as henna), temporary dyes (water-soluble dyes that withstand only one-time shampooing), semi-permanent dyes (which can withstand 4-5 times of shampooing) and permanent hair colors.[2] Permanent hair colors are the most popular hair dye products. They may be further divided into oxidation hair dyes and progressive hair dyes. Oxidation hair dye products consist of (1) a solution of dye intermediates, e.g., p-phenylenediamine, which form hair dyes on chemical reaction, and preformed dyes, e.g., 2-nitro-p-phenylenediamine, which already are dyes and are added to achieve the intended shades, in an aqueous, ammoniacal vehicle containing soap, detergents and conditioning agents; and, (2) a solution of hydrogen peroxide, usually 6%, in water or a cream lotion.

The ammoniacal dye solution and the hydrogen peroxide solution, often called the developer, are mixed shortly before application to the hair. The applied mixture causes the hair to swell and the dye intermediates (and preformed dyes) penetrate the hair shaft to some extent before they have fully reacted with each other and the hydrogen peroxide and formed the hair dye.

Progressive hair dye products contain lead acetate as the active ingredient. Lead acetate is approved as a color additive for coloring hair on the scalp at concentrations not exceeding 0.6% w/v, calculated as metallic lead . Bismuth citrate, the other approved color additive, is used to a much lesser extent. Progressive hair dyes change the color of hair gradually from light straw color to almost black by reacting with the sulfur of hair keratin as well as oxidizing on the hair surface.

Hair dye consumption is not an uncommon means of intentional self-harm. It has been reported around the world, more so in the underdeveloped and developing countries. Case series from Khartoum[3,4] and Casablanca[5] have reported 46 cases of hair dye (paraphenylenediamine) poisoning. Paraphenylenediamine poisoning was the number one cause of poisoning in Morocco during the 1990s.[6] Numerous case reports have been reported from India.[7–10]

## PATHOGENESIS

Paraphenylene diamine *(PPD)*: It is a coal-tar derivative, which on oxidation produces Bondrowski's base, which is allergenic, mutagenic and highly toxic. Poisoning with PPD presents with the characteristic features of severe angioneurotic edema, rhabdomyolysis and intravascular hemolysis with hemoglobinuria culminating in acute renal failure.

The mechanism of rhabdomyolysis has been investigated in rats by Yabe K.[11] PPD can bring about rhabdomyolysis by promoting calcium release and leakage of calcium ions from the smooth endoplasmic reticulum, followed by continuous contraction and irreversible change in the muscle's structure. Rhabdomyolysis is the main cause of acute renal failure and the morbidity and mortality are high once renal failure develops. Hypovolemia and the direct toxic effects of PPD or its metabolites on the kidneys also contribute. The respiratory syndrome following the ingestion of PPD is represented by asphyxia and respiratory failure secondary to inflammatory edema involving cricopharynx and larynx. Histologic changes of acute tubular necrosis have been described in PPD poisoning.[12] Myoglobinuric ARF is observed in the tropics after a variety of poisonings with agents such as mercuric chloride, zinc and aluminum phosphide. The diagnosis is established by the demonstration of myoglobin in urine and elevated levels of creatine phosphokinase and aldolase in the serum. Since myoglobin is a small molecule with a molecular weight of 17 kDa, which binds only lightly to the plasma proteins, it escapes easily in the urine. Therefore, the urine may not contain myoglobin if the patient presents late in the course of the disease and the true incidence of myoglobinuric ARF will be underestimated. Severe hypocalcemia and hyperuricemia during the oliguric phase and hypercalcemia during the diuretic phase are characteristic of this condition. The pathogenesis of myoglobinuric ARF is similar to that following intravascular hemolysis. Renal histology shows acute tubular necrosis. Early recognition of Rhabdomyolysis is crucial since intravenous bicarbonate and saline have been shown to ameliorate the development of acute renal failure in crush injuries. PPD has also been shown to produce myocarditis and arrhythmias leading to sudden death.

Propylene glycol, one another potential nephrotoxin is a viscous, colorless liquid commonly used as a solvent in hair dyes. It is associated with hyperosmolality, raised anion gap metabolic acidosis, central nervous system depression, arrhythmias and renal dysfunction.[13] Acute tubular necrosis has been described. Proximal renal tubular cell swelling and vacuole formation have also been seen in propylene glycol ingestion.[14] But the characteristic features of rhabdomyolysis and laryngeal edema which typify PPD poisoning are absent. Resorcinol found in hair dyes is a phenol derivative, which may also contribute to renal toxicity. In addition, a few hair dyes also contain lead acetate and Bismuth sulfate, which can cause chronic kidney disease or acute interstitial nephritis respectively.

## CLINICAL MANIFESTATIONS

The characteristic triad of features encountered are early angioneurotic edema with stridor, rhabdomyolysis with chocolate colored urine and acute renal failure. When ever this combination occurs in poisoning, hair dye is a strong suspect.

Kallel *et al.*, studied 19 patients with PPD intoxication in Tunisia over a 6-year period.[15] Clinical symptoms were dominated by cervicofacial edema (79%), chocolate-brown colored urine (74%), upper airway tract edema (68.4%), oliguria (36.8%), muscular edema (26.3%) and shock (26.3%). Rhabdomyolysis and metabolic acidosis were seen in all the patients. ARF was seen in 47.4% and hyperkalemia in 26.3%. In the series by Ram *et al.*, the incidence of ARF was 70%.[8] All had rhabdomyolysis. Four patients required tracheostomy and ventilatory assistance. Two large series have been published from the African continent. In a 11-year retrospective study of PPD poisoning reported to the Poison control centre of Morocco, 374 cases were analysed. Rhabdomyolysis and acute renal failure were the main contributing factors for the 21% mortality. Suliman *et al.*, studied 150 patients who presented with PPD poisoning in Sudan over a 10-year period. Sixty percent had ARF requiring dialysis whereas 30% had ARF which recovered with conservative measures[16] Angioneurotic edema was encountered in 68% and emergency tracheostomy had to be done in 15.8%. All patients recovered renal function after a mean period of 15 days of dialysis. Interestingly renal biopsy was undertaken after recovery of renal function in 20 patients. Not surprisingly, the histology was normal in almost all cases.

In another as yet unpublished observational study of hair dye poisoning in Hyderabad, India, acute renal failure developed in 100% of patients due to rhabdomyolysis.[17] The mortality was high. Six out of the ten patients died (60%). Amount of poison ingested, hyperkalemia, hypocalcemia and hyperphosphatemia were predictive of mortality.

## TREATMENT

Hair dye ingestion is a medical emergency. Emergency measures should include gastric lavage. Patients should be monitored for respiratory distress and endotracheal intubation has to be performed early if laryngeal edema develops. Metabolic acidosis has to be corrected. Early intervention with half normal saline and soda bicarbonate infusion has been shown to be beneficial in Rhabdomyolysis. All modalities of dialysis – hemodialysis, peritoneal dialysis and continuous renal replacement therapy have been tried and have been found to be useful in acute renal failure. Mortality rates vary between 0.03% and 60%.

## CONCLUSIONS

Whenever the characteristic triad of stridor due to upper airway edema, rhabdomyolysis and acute renal failure develops in a poisoning, hair dye should be considered. Early airway protection, alkaline diuresis and dialysis are the three management strategies helpful in this situation. Awareness about this condition is helpful in early intervention to reduce mortality.
